# Recombination landscape and karyotypic variations revealed by linkage mapping in the grapevine downy mildew pathogen *Plasmopara viticola*

**DOI:** 10.1093/g3journal/jkae259

**Published:** 2024-12-02

**Authors:** Etienne Dvorak, Isabelle D Mazet, Carole Couture, François Delmotte, Marie Foulongne-Oriol

**Affiliations:** SAVE, INRAE, Bordeaux Sciences Agro, ISVV, Villenave d’Ornon F-33140, France; SAVE, INRAE, Bordeaux Sciences Agro, ISVV, Villenave d’Ornon F-33140, France; SAVE, INRAE, Bordeaux Sciences Agro, ISVV, Villenave d’Ornon F-33140, France; SAVE, INRAE, Bordeaux Sciences Agro, ISVV, Villenave d’Ornon F-33140, France; MYCSA, INRAE, Villenave d’Ornon F-33140, France

**Keywords:** plant pathogen, genetic map, meiotic recombination, karyotypic anomalies

## Abstract

*Plasmopara viticola*, the causal agent of grapevine downy mildew, is a biotrophic oomycete engaged in a tight coevolutionary relationship with its host. Rapid adaptation of the pathogen is favored by annual sexual reproduction that generates genotypic diversity. With the aim of studying the recombination landscape across the *P. viticola* genome, we generated 2 half-sibling F1 progenies (*N* = 189 and 162). Using targeted SNP sequencing, between 1,405 and 1,894 markers were included in parental linkage maps, and a consensus map was obtained by integrating 4,509 markers. The reference genome could be assembled into 17 pseudochromosomes, anchoring 88% of its physical length. We observed a strong collinearity between parental genomes and extensive synteny with the downy mildew *Peronospora effusa*. In the consensus map, the median recombination rate was 13.8 cM/Mb. The local recombination rate was highly variable along chromosomes, and recombination was suppressed in putative centromeric regions. Recombination rate was found negatively correlated with repeats’ coverage and positively correlated with gene coverage. However, genes encoding secreted proteins and putative effectors were underrepresented in highly recombining regions. In both progenies, about 5% of the individuals presented karyotypic anomalies. Aneuploidies and triploidies almost exclusively originated from the male-transmitted chromosomes. Triploids resulted from fertilization by diploid gametes, but also from dispermy. Obligatory sexual reproduction each year may explain the lower level of karyotypic variation in *P. viticola* compared to other oomycetes. The linkage maps will be useful to guide future de novo chromosome-scale assemblies of *P. viticola* genomes and to perform forward genetics.

## Introduction

Filamentous plant pathogens and their hosts are engaged in coevolution relationships, in which reciprocal selection takes place (Brown and Tellier 2011). As plants adapt to avoid or limit infection, pathogens counter-adapt to overcome their defenses. This has crucial implications for crop production systems, in which disease control by resistant cultivars can quickly lose efficiency. The evolutionary potential of a plant pathogen depends on its mutation rate, its effective population size, its gene flow capacity, and its mode of reproduction (McDonald and Linde 2002). Among these forces, the prevalence of sexual reproduction plays an important role, because recombination creates original allele associations and leads to an increased genotypic diversity on which natural selection can act. In addition, meiosis itself can generate structural rearrangements and karyotypic variability, which are common in plant pathogens and can have a strong impact on life history traits (Mehrabi *et al.* 2017). In agricultural pathosystems, sexual reproduction can be involved in the emergence of novel pathotypes adapted to resistant cultivars (Ahmed *et al.* 2012) or different host species (McMullan *et al.* 2015; Menardo *et al.* 2016).

Filamentous plant pathogen genomes contain a large number of genes encoding secreted proteins that promote colonization by altering host physiology and suppressing immune responses (Franceschetti *et al.* 2017). These genes tend to be located in regions rich in repetitive elements. This observation led to the concept of the two-speed genome model, according to which repeat-rich and gene-sparse compartments provide a genomic environment that fosters the fast evolution of genes crucial for host adaptation (Dong *et al.* 2015). In parallel, fine-mapping of crossing-overs (COs) in fungal pathogens showed that genes involved in host–pathogen interaction are preferentially located in highly recombining regions (Croll *et al.* 2015; Laurent *et al.* 2018). This points to a tight link between the functional architecture and the recombination landscape of these genomes.

In regions where COs occur frequently, linkage disequilibrium is reduced, which locally increases the efficacy of selection and could therefore boost the speed of pathogen adaptation. Compared to fungal plant pathogen, recombination data remain scarce for oomycetes. Linkage maps have been produced with increasing resolution for several major oomycete pathogens (Hulbert *et al.* 1988; Van der Lee *et al.* 1997; Lamour *et al.* 2012; Matson *et al.* 2022), but these studies did not focus on recombination per se.

Oomycetes present strong convergence in terms of morphology and lifestyle with true fungi, but they are phylogenetically distant. Their distinctive features include biflagellate zoospores, little to no chitin in cell walls, the absence of an haploid stage, and large sexual propagules called oospores. Polyploidy and aneuploidy are frequent in several species, which can complicate genetic analyses (Kasuga *et al.* 2016; Li *et al.* 2017). Plant pathogenic oomycetes display a wide range of modes of reproduction, with diverse rates of sexual reproduction (Judelson 2009). The prevalence of outcrossing also differs between species, as some species are homothallic and reproduce predominantly by selfing (Judelson 2009). Plant pathogenic oomycete genomes encode hundreds of putative effectors that are often characterized by amino acid sequence motifs such as RXLR/dEER or LXLFLAK (Schornack *et al.* 2009). They typically occur in large clusters in the genome, suggesting that they evolve by tandem gene duplication (Schornack *et al.* 2009). It has been suggested that chromosomal rearrangements through unequal COs and/or transposon activity mediate the expansion of RXLR effector families (Matson *et al.* 2022).

*Plasmopara viticola*, the causal agent of grapevine downy mildew, is a major oomycete pathogen threatening vineyards worldwide (Gessler *et al.* 2011; Kamoun *et al.* 2015). It has demonstrated a high ability to adapt by overcoming resistance genes (Wingerter *et al.* 2021; Paineau *et al.* 2022) and losing fungicide sensitivity (Chen *et al.* 2007; Delmas *et al.* 2017). This high evolutionary potential relies on large population sizes, airborne dispersal, and a mixed reproduction system (McDonald and Linde 2002). At the field level, *P. viticola* populations are panmictic and highly diverse (Gobbin *et al.* 2006), with a high heterozygosity rate (Dussert *et al.* 2019). This is favored by outcrossing, as the species is heterothallic with 2 mating types denominated P1 and P2 (Wong *et al.* 2001). Self-incompatibility is controlled by a 570-kb locus exhibiting 2 highly divergent alleles, with P2 strains being homozygous (MAT-a/MAT-a) and P1 strains heterozygous (MAT-a/MAT-b) (Dussert *et al.* 2020b). As *P. viticola* is a strictly biotrophic pathogen, sexual reproduction takes place inside the grapevine mesophyll. There, strains of opposite mating types form gametangia in which meiosis occurs shortly before fertilization. Female oogonia are fertilized by male antheridia and mature into mononucleated oospores, each containing a distinct diploid zygote (Vercesi *et al.* 1999). Oospores are the only overwintering form under temperate climates. Consequently, the life cycle is characterized by mandatory annual sexual reproduction in the main grape growing areas. Original genotypes are thus generated each year through recombination. An in-depth comprehension of this key driver of the *P. viticola* genome evolution will contribute to a more effective and sustainable management of the disease.

In this study, we assess the relation between meiotic recombination and the genome architecture of an oomycete plant pathogen. We build a set of unprecedented high-density linkage maps based on targeted genotyping by sequencing of 2 large *P. viticola* progenies. We compare the maps obtained for 3 different parent strains and combine data from the 2 half-sibling families to obtain a consensus map. Thanks to the linkage data, we generate a pseudoassembly of the reference genome, making it possible to conduct analyses at the chromosome scale. We compare the local recombination rate along the reconstructed chromosomes with various genomic elements, such as repeats and putative effector genes. We also assess the extent of karyotypic variation in the offspring and identify multiple mechanisms of origin for these anomalies. These results are discussed in light of the evolutionary potential of *P. viticola*.

## Materials and methods

### Generation of the progenies

**Fig. 1.**
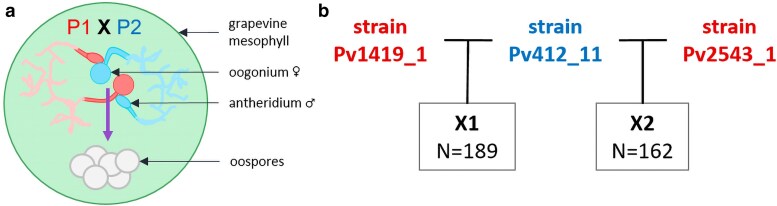
Controlled sexual reproduction of *P. viticola*. a) Gamete-producing organs of opposite mating types P1 and P2 meeting inside grapevine leaf disk tissues. Fertilized oogonia mature into resting thick-walled oospores. b) Crossing plan carried out in this study. The 2 crosses have 1 parent in common, therefore yielding 2 half-sibling progenies, designated X1 and X2.

As *P. viticola* is an obligate biotrophic organism, all strains used in the study were grown using leaf tissues sampled from *Vitis vinifera* cv. ‘Cabernet-Sauvignon’ cuttings cultivated in pots under greenhouse conditions. Sexual reproduction was carried out in planta via coinoculation of strains of opposite mating types (Fig. 1a). We used single-sporangia strains that were previously characterized by Paineau *et al.* (2022): Pv412_11 (P2) was collected in Switzerland (2010), Pv1419_1 (P1) in Germany (2012), and Pv2543_1 (P1) in Hungary (2013). We generated 2 progenies with 1 parent (Pv412_11) in common between the 2 crosses (Fig. 1b).

Disks were excised from the seventh leaf from the apex and coinoculated with the 2 parents as described in Dussert *et al.* (2020b). Leaf disks in which oospores were observed were put under maturation conditions in the dark at 4°C for 8–10 months. Leaf disks were then ground in a glass potter, and oospores were retrieved using nylon filters (protocol adapted from Toffolatti *et al.* 2007). Two steps of filtration were performed to eliminate larger debris (pore size 100 and 60 µm). A third step was done to filter smaller debris while retaining oospores on the filter (pore size 20 µm). The oospores were resuspended in sterile water with an adjusted concentration of 6–7 per microliter, and 15 µL droplets was deposited on 1% water agar plates. These were placed in a growth chamber for 7–12 days at 22°C in the dark to trigger germination. The plates were checked daily for macrosporangia formation under a binocular magnifier. Germinating oospores were retrieved one by one with a pipette and inoculated on a disk excised from the fourth leaf from the apex. After successful infection, asexual sporangia were retrieved from the surface of the leaf disk. A step of single sporangium isolation was performed to further ensure that the recovered strains corresponded to a unique genotype, as described in Paineau *et al.* (2022). In total, 189 offspring were obtained for X1 and 162 for X2 (Fig. 1b). Sporulating leaf fragments were desiccated and then stored at −20°C until further use.

### DNA extraction

Strains were multiplied on 3 detached grapevine leaves (fourth leaf from the apex), and pellets of sporangiophores and sporangia were collected and treated as described in Dussert *et al.* (2019). DNA was extracted following a CTAB extraction protocol adapted from Möller *et al.* (1992). In particular, protein degradation was performed using 10 µL of Thermo Scientific Proteinase K (20 mg/L, product reference: EO0491). A step of RNA removal was performed after CTAB lysis by adding 10 µL of Qiagen Ribonuclease A (catalog number: 19101) diluted 10-fold, followed by incubation at 37°C for 30 min. DNA purity and concentration were assessed using respectively a DeNovix DS-11 spectrophotometer and a Qubit 3 fluorometer (Invitrogen).

### SNP genotyping

Parent strains were sequenced to identify variants suitable for use as genetic markers. Paired-end sequencing was performed at the Genoscope, CEA—Institut de biologie François Jacob (Evry, France) with 2 runs of Illumina MiSeq 2 × 250 bp. Following the pipeline used in Paineau *et al.* (2024), variants were called with GATK HaplotypeCaller v4.2.6.1 (McKenna *et al.* 2010). A total of 3,543,322 potential polymorphic sites were called on the Pv221 reference genome assembly (Dussert *et al.* 2019).

#### Definition of the set of targeted markers

In order to find high-confidence SNPs that would segregate in the progenies, the vcf file obtained was subjected to a series of filters. Repeated sequences were masked using bedtools v2.27.1 (Quinlan and Hall 2010), following the annotation available in Dussert *et al.* (2020a). Variants with missing data in any of the parent strains, indels, and multiallelic SNPs were ignored. After examination of the distribution of GATK annotation values, biallelic SNPs were selected using GATK VariantFiltration command with the following parameters: QD <5, QUAL <30, DP <30, SOR >3, FS >40, MQ <50, MQRankSum <-5, ReadPosRankSum <-5.

To minimize the odds of incorrect calling due to mapping errors, SNPs were considered reliably heterozygous for an individual if the minor allele frequency (MAF) was superior to 0.4 and homozygous if MAF <0.1. Other SNPs were ignored. After all the steps above, 1,200,172 SNPs were retained.

In our setting, SNPs were informative if they were expected to segregate in the F1 progeny in a 1:1 or 1:2:1 ratio. For each cross, we considered SNPs heterozygous in one parent and homozygous in the other (testcross configuration) and SNPs heterozygous in both parents (intercross configuration). Furthermore, we aimed to define a unique set of markers segregating in the 2 F1 progenies, which meant SNPs had to be heterozygous in at least one parent for each cross. We preferentially selected markers in the first 3 configurations described in Table 1.

**Table 1.** Informative marker configurations and their expected segregation patterns.

Genotype configuration	Pv1419_1	Pv412_11	Pv2543_1	Segregation in X1	Segregation in X2
1	H	A	H	1:1	1:1
2	A	H	H	1:1	1:2:1
3	H	H	A	1:2:1	1:1
4	A	H	A	1:1	1:1
5	H	H	H	1:2:1	1:2:1

A, homozygous; H, heterozygous.

A minimal distance of 6.5 kb between 2 markers on the same scaffold was chosen. Potential marker positions were then sent to the sequencing facility for the design of specific pairs of probes. Finally, 4,996 genomic markers were selected to include as many scaffolds as possible, to which we added 4 mitochondrial markers.

#### Targeted genotyping by sequencing

Parent strains and their offspring were genotyped by targeted sequencing at the INRAE EPGV facility (National Genotyping Center, Evry, France) using single primer enrichment technology (SPET) (Scaglione *et al.* 2019) commercialized under the name Allegro. This method is based on a single primer extension reaction to perform multiplex enrichment of a set of thousands of target loci. SPET probes are around 40 bases long and are designed adjacent to a region containing a target variant. This enables the genotyping of the target SNPs but also of additional ones that may surround it. We thereafter refer to these additional variants as flanking variants. Libraries were prepared using the Tecan Allegro Targeted Genotyping V2 kit, with 48 samples pooled per library. One parental genotype was included in all libraries as a control. Sequencing was performed on an Illumina NextSeq 550 (2 × 150 bp paired-end reads).

#### Calling of targeted SNPs and flanking variants

Reads were mapped on the PV221 reference genome (Dussert *et al.* 2019) with bwa-mem v2.2.1 (Vasimuddin *et al.* 2019). Duplicates were removed with the GATK command MarkDuplicatesSpark. Reads with mapping quality <20 were filtered. Probe sequences in the reads were tagged using Tecan Genomics’ tool ProbeFilter (available at https://interval.bio/allegro_bioinformatics.html) so that they could be ignored downstream. Variant calling was done using GATK HaplotypeCaller in GVCF mode. Then, GVCFs were consolidated scaffold by scaffold using GATK GenomicsDBImport, before joint calling of genotypes with GATK GenotypeGVCFs. Finally, all files were merged using GATK GatherVcfs. Variants with read depth (DP) < 10 were set to missing data. Additionally, they were considered heterozygous only if MAF >0.1.

#### Mitochondrial heredity

In oomycetes, mitochondria are solely transmitted by female oogonia that provide the cytoplasm of the future oospores (Martin 1989; Judelson 2009). Four mitochondrial markers were used to follow this heredity and determine the female parent of each offspring.

### Linkage map construction

As *P. viticola* is a highly heterozygous self-incompatible organism (Wong *et al.* 2001; Dussert *et al.* 2019), we used a 2-way pseudotestcross mapping strategy. Heterozygous markers in each parent were used to build distinct parental maps, with each parent acting as a “tester” for the other (Hulbert *et al.* 1988; Grattapaglia and Sederoff 1994).

#### Parental maps

Parental linkage maps were independently built using r/ASMap v1.0.4 (Taylor and Butler 2017) in successive steps. Individuals with excessive heterozygosity rates (>0.6) were ignored, as this could be due to genotype mixture or polyploidy. The presence of identical genotypes was checked (>95% marker identity). The marker phase in each parent was initially unknown, so all markers were added twice, once for each opposite phase, as mentioned in Brelsford *et al.* (2016). Duplicate linkage groups (LGs) were subsequently removed. Markers were pulled out when more than 20% of the genotypes were missing or if their segregation pattern was significantly distorted (*χ*^2^ test, *P* < 0.1/number of markers).

First of all, framework maps were constructed with only testcross target markers (homozygous in one parent and heterozygous in the other, as described in Table 1). Testcross flanking markers were added to each map if they belonged to scaffolds with little or no markers already positioned. A few intercross (heterozygous in both parents) target markers were incorporated to densify the maps where long gaps remained. For these, 0/1 genotypes were set as missing because of the impossibility of knowing the parental origin of the alleles. Consequently, the missing data threshold was bypassed for these markers. Two LGs of the X1 maps exhibited large gaps because of the removal of closely linked markers that were moderately distorted. We decided to put back these groups of markers as they allowed to complete the maps without aberrant behavior (see *Results*).

One marker per unique segregation pattern was retained for map construction with the mstmap function. LGs were separated using a LOD threshold of 8. Genetic distances were calculated with the Kosambi mapping function. After the initial marker ordering, distances were recomputed by applying a genotyping error rate of 0.5%, in accordance with the repeatability of the control samples. Then, the maps were manually curated by using the compareorder function. When contradictions were observed between genetic and physical orders, markers were switched if doing so increased likelihood and reduced LG length. Finally, cosegregating markers were added back.

#### Consensus linkage map

Scaffolds were ordered and oriented by combining information from the 4 final parental maps with ALLMAPS (Tang *et al.* 2015). As there were very few discrepancies between genetic and physical order (Spearman's *ρ* ranging from 0.98 to 1.00), markers from all maps were ordered according to their physical position in the reference genome. In order to integrate linkage information from both families, the consensus map was built using Lep-MAP3 (version available on 2023 August 20) (Rastas 2017). To avoid the spurious estimation of recombination fraction between markers flanking the same sequenced target, only the most informative ones were kept for each targeted region (i.e. markers for which there were as many heterozygous parents as possible). The genotypes of the parents and the 2 half-sib progenies were determined using the ParentCall2 command with option halfSibs=1. Markers were phased, and the genetic distances were calculated for each LG with the Kosambi mapping function and a genotyping error rate of 0.5%. This was achieved using the OrderMarkers2 command with options improveOrder=0, useKosambi=1, minError=0.005, sexAveraged=1, outputPhasedData=1.

### Genomic features

#### Synteny

The synteny between the anchored *P. viticola* scaffolds and the chromosome-level assembly of *Peronospora effusa* was assessed using SynMap2 (Haug-Baltzell *et al.* 2017). Coding sequence positions were retrieved from the published annotations (Dussert *et al.* 2019; Fletcher *et al.* 2022) and were aligned with the “LAST” algorithm (default parameters).

#### Centromere identification

Putative centromeric regions were identified by the presence (BLASTn alignment length >500 bp) of a *Phytophthora sojae* copia-like transposon proposed as a distinctive feature of oomycete centromeres (Fang *et al.* 2020). The regions were confirmed via synteny with the *P. effusa* centromeres described by Fletcher *et al.* (2022).

#### Pseudoassembly and annotations

Thanks to the linkage data, the scaffolds of the reference genome were joined and oriented using ALLMAPS, adding 100 *N*s between each one. Annotations from Dussert *et al.* (2019) were lifted over to this pseudoassembly, including the positions of predicted secreted protein genes and putative effector genes.

#### Estimation of the recombination rate

Using genetic distances from the consensus map, the recombination rate in centimorgan per megabase was approximated in the pseudoassembly using locally estimated scatterplot smoothing implemented in r/MareyMap v1.3.6 (Rezvoy *et al.* 2007), with parameters span=0.1 and degree=2. The estimation was retrieved every 10 kb for graphical representation and statistical analyses.

#### Gene enrichment analysis

Ten-kilobase genomic bins for which recombination rate could be estimated were regrouped by deciles. Limits were calculated using the quantile() function implemented in R v1.4.2. Each decile was tested for enrichment or depletion in secreted protein and putative effector genes (hypergeometric test, *α* = 0.05).

### Identification of karyotypic anomalies

Aneuploid and triploid individuals were first identified by their highly excessive number of apparent COs in one or several LGs of a parental map. For each individual, the number of detected COs in each parental LG was obtained using the countXO function of r/qtl v1.60 (Arends *et al.* 2010). These observations were supported by the examination of allelic ratios in relevant LGs or the entire genome. Aneuploidies and partial duplication/deletions could be additionally corroborated by comparing reads’ mapping coverage between LGs. The affected parental linkage map designated from which parent the anomaly originated. The sequencing depth of the targeted SNPs was retrieved from the reads aligned to the reference genome using mosdepth v.0.2.5 (Pedersen and Quinlan 2018). For partial duplication/deletion and copy-neutral losses of heterozygosity, the estimated boundaries correspond to the region with continuous switching of markers from one phase to another (as mentioned in Lamour *et al.* 2012). Such a switch indicates that a portion of the chromosome is in fact fully homozygous or fully heterozygous for the considered markers.

Aneuploid strains were kept for the construction of linkage maps as they still provided valuable information for most of the genome. The false recombination events in affected LGs had little impact thanks to the allowance for 0.5% of genotyping errors. Triploids were all put aside previously because of their excessive heterozygosity, as mentioned above.

### Assessment of origin of triploidy

To identify the most likely mechanism of origin of triploidy for each individual, we analyzed the allelic composition of the 2 sets of chromosomes transmitted by only one parent. Specifically, we determined if parental heterozygosity was maintained (nonreduction) or reduced to homozygosity in pericentromeric markers, as they do not recombine. By combining observations on multiple chromosomes, we could consider and rule out certain mechanisms for each individual, as explained in Zaragoza *et al.* (2000).

Failure of disjunction in meiosis I is characterized by nonreduced pericentromeric markers and failure of disjunction in meiosis II by consistently reduced markers. Fertilization of 1 female gamete by 2 haploid male gametes, known as dispermy, leads to a random mixture of both. Consequently, we reconstructed the alleles transmitted by each parent for pericentromeric markers in the LGs in which putative centromeres were confidently identified. The number of copies of each allele was estimated using the allelic ratios of the markers. For example, let us consider a marker for which parent 1 (genotype AA) passed on 1 copy and parent 2 (AB) 2 copies. If the observed genotype of the offspring is AA, then parent 2 necessarily transmitted 2 “A” copies. When the observed genotype is AB, there are 2 possibilities: (1) if the proportion of “A” is statistically equal to 2/3, we can infer that parent 2 transmitted one “A” copy and one “B” copy; (2) if this proportion corresponds instead to 1/3, then it means that 2 “B” copies were passed on. The proportions were tested using a *χ*^2^ test with *α* = 0.05.

## Results

### Targeted genotyping by sequencing of informative SNPs

Two half-sibling *P. viticola* progenies were genotyped to build linkage maps based on the recombination of heterozygous markers during parental meiosis. To do so, strains were genotyped by targeted sequencing of informative nuclear markers. Out of the 4,996 targeted regions, 4,990 were successfully sequenced, but 2 markers appeared triallelic and were therefore ignored. The mean sequencing depth of the targets was 173x (minimum 51x, maximum 506x). The repeatability of the genotyping of target SNPs in the control samples was 99.4%. Individuals were successfully genotyped for 97% of all SNPs on average (minimum 91%). The Allegro technology provides sequences up to a few hundred bases upstream and downstream of the target SNPs. Thus, a total of 75,812 potentially informative flanking variants were called in X1 and 41,215 in X2. Overall, 4,988 high-confidence target SNPs were initially available for the construction of the linkage maps, to which 781 flanking variants were added to reduce potential gaps.

### Uniparental inheritance of mitochondria

Beside nuclear SNPs, we followed the inheritance of 4 mitochondrial markers. As expected, mitochondria were uniparentally inherited. Both parents in each cross could transmit it, demonstrating that each strain produced male and female gametes. However, the sex contributions were uneven in X1 (Pearson's *χ*^2^ test, *P* = 1.2e^−4^), in which Pv1419_1 was the female parent for two-thirds (64.0%) of the offspring. No such imbalance was observed in X2 (*P* = 0.43).

### Construction of a set of parental and consensus linkage maps

#### Markers are consistently gathered into 17 LGs

One linkage map for each parent of each cross was built using primarily testcross target markers (segregating 1:1). Between 1,405 and 1,894 markers were positioned depending on the parent strain considered (Table 2). A few intercross markers (segregating 1:2:1) were also incorporated (minimum 5 and maximum 28).

**Table 2.** Features of the 4 parental linkage maps and the consensus map.

Map	Pv412_11 X1	Pv412_11 X2	Pv1419_1 X1	Pv2543_1 X2	Consensus
Linkage groups	17	17	17	17	17
Markers	1,405	1,894	1,879	1,578	4,509
Markers with unique segregation pattern	717	842	946	728	2,739
Length (cM)	1,167.5	1,221.7	2,073.4	1,379.2	1,473
Average spacing (cM)	0.8	0.7	1.1	0.9	0.3
Maximum spacing (cM)	13.4	11.4	18.8	17.3	5.8
Anchored scaffolds	144	142	143	120	154
Total anchored length (Mb)	80.13 (86.2%)	79.93 (86.0%)	80.33 (86.4%)	76.19 (82.0%)	81.64 (87.8%)

Each map is composed of 17 LGs (Table 2), which correspond in all likelihood to the number of chromosome pairs. The lengths of the 2 Pv412_11 maps were similar (1,167.5 cM in X1 vs 1,221.7 cM in X2) and a bit shorter than the Pv2543_1 map (1,379.2 cM). The Pv1419_1 map stands out as much longer (2,073.4 cM), with a higher number of unique segregation patterns (946 vs 717–842) (Table 2). The average spacing between markers ranged from 0.7 cM in Pv412_11-X2 to 1.1 cM in Pv1419_1-X1. Some moderate gaps remained, but they were located on different LGs depending on the map (see Supplementary Figs. 1–3 for other LGs). For example, the largest gap in Pv412_11-X2 (11.4 cM) was located in LG6, while it was found in LG12 in the Pv1419_1-X1 map (18.8 cM) (Table 2).

#### Two regions exhibit distortion of segregation in 1 cross

Markers with biased segregation ratios were initially not included in the maps (*χ*^2^ test, *α* < 0.1/number of markers). This included 54 SNPs in Pv412_11-X1, 18 in Pv1419_1-X1, 8 in Pv412_11-X2, and 6 in Pv2543_1-X2. In the 2 X1 maps, most distorted markers were linked and located in 2 different LGs. We added these markers back to avoid large gaps in these LGs (39 markers in Pv412_11-X1, 17 markers in Pv1419_1-X1). In these regions, markers segregated approximately at a 1:2 frequency instead of the expected 1:1 ratio. Each region was actually affected in only 1 parental map, and the corresponding intervals were free from distortion in the other map. They were located on LG2 in the Pv1419_1 map and LG13 on the Pv412_11 map. The corresponding physical segments stretched over 0.94 and 1.99 Mb, respectively (Supplementary Fig. 4). Notably, the LG13 region was not distorted in the Pv412_11 map obtained in the other cross. The 2 loci were not in linkage disequilibrium in the X1 progeny. There was no association with mitochondrial markers either. Thus, the 2 segregation distortion events appear independent.

#### The parental genomes are highly collinear

**Fig. 2.**
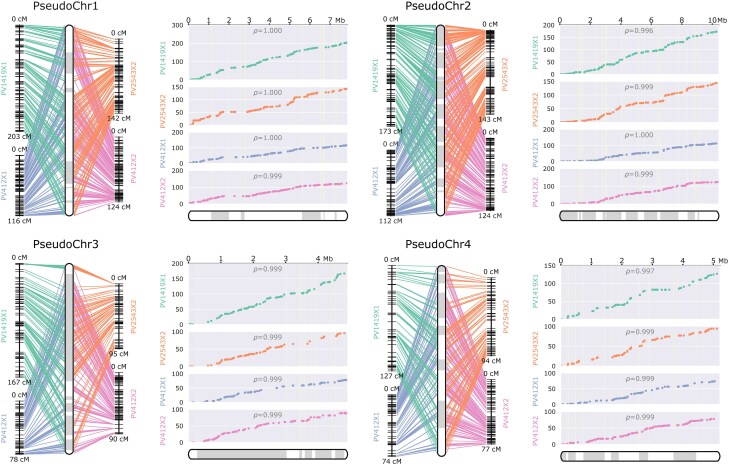
*Plasmopara viticola* parental linkage maps. The first 4 LGs are shown (see Supplementary Figs. 1–3 for the other LGs). The scaffolds of the reference assembly were anchored and oriented thanks to the linkage information from the 4 parental maps. They were joined together as pseudochromosomes, which are represented at the center of the left panel and the bottom of the right panel. The scaffolds’ limits are signaled by the switch between white and gray sections. Left panels: correspondence between the physical positions and the genetic positions in the LGs of the 4 parental maps. Right panels: genetic positions (*y*-axis) vs physical positions (*x*-axis). A strong slope implies a highly recombining region, whereas a rather flat line indicates little or no recombination in the interval. The Spearman *ρ* measures the rank correlation between the genetic and the physical order for each LG. Plots were generated using ALLMAPS.

The parental maps were remarkably collinear between them and with the reference genome (correlation of genetic and physical order *ρ* ≥ 0.98) (Fig. 2). No large-scale structural variations were observed at the resolution level of the linkage maps.

#### The recombination activity varies between strains

The recombination patterns appear similar between all parental maps as can be seen in the right panels of Fig. 2. Interestingly, much more recombination events were detected in Pv1419_1 meiosis than in other parents (from 51 to 80% more COs on average) (Supplementary Fig. 5). This is reflected by its inflated linkage map length and a higher average spacing between markers (Table 2). The increase in recombination was evenly spread across all LGs (Fig. 2).

The number of COs between maternal and paternal meiosis slightly differed for all parents, although not always in the same direction. More COs occurred in female meiosis for Pv412_11 and Pv2543_1 (+8.0 to 15.6% compared to male meiosis, *P* < 0.034), but the opposite was observed for Pv1419_1 (−10.4%, *P* = 0.0018) (Supplementary Fig. 5).

#### The consensus linkage map covers most of the genome length

**Fig. 3.**
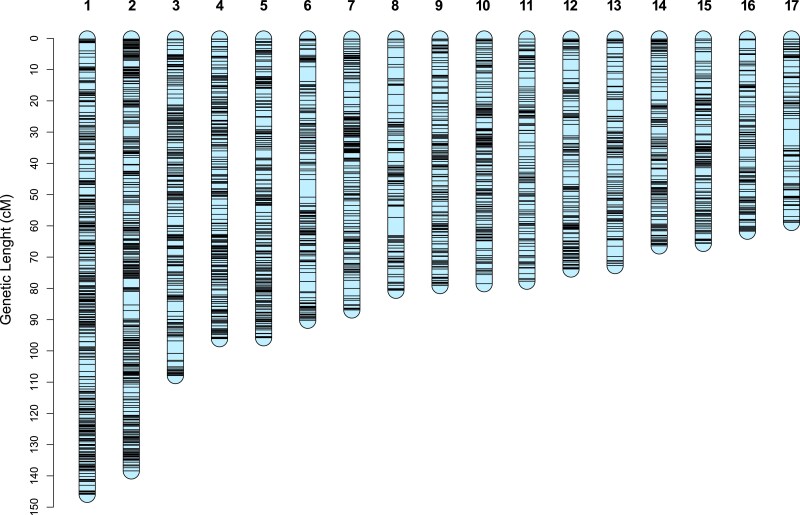
Genetic length and marker distribution of the 17 LGs in the consensus linkage map of *P. viticola*. The scale on the left indicates the genetic distances in centimorgan determined by the Kosambi mapping function. Plot made using r/LinkageMapView v2.1.2.

We took advantage of the high collinearity between the parental genomes to build a consensus linkage map integrating the genotyping data of the 2 offspring. As desired initially, most markers (97.6%) were informative in both crosses, ensuring a good accuracy in the computing of genetic distances. This resulted in the positioning of 4,509 markers (2,739 unique positions) (Fig. 3), with a reduced spacing between markers and much shorter gaps. In total, 154 scaffolds representing 87.8% of the genome length were anchored in a single map (Table 2). The rest of the genome consists of small repeat-rich scaffolds on which SPET probes were difficult to design. The LGs were numbered from the longest to the shortest according to their genetic length in this consensus map.

#### The LGs are highly syntenic with the *P. effusa* chromosomes

**Fig. 4.**
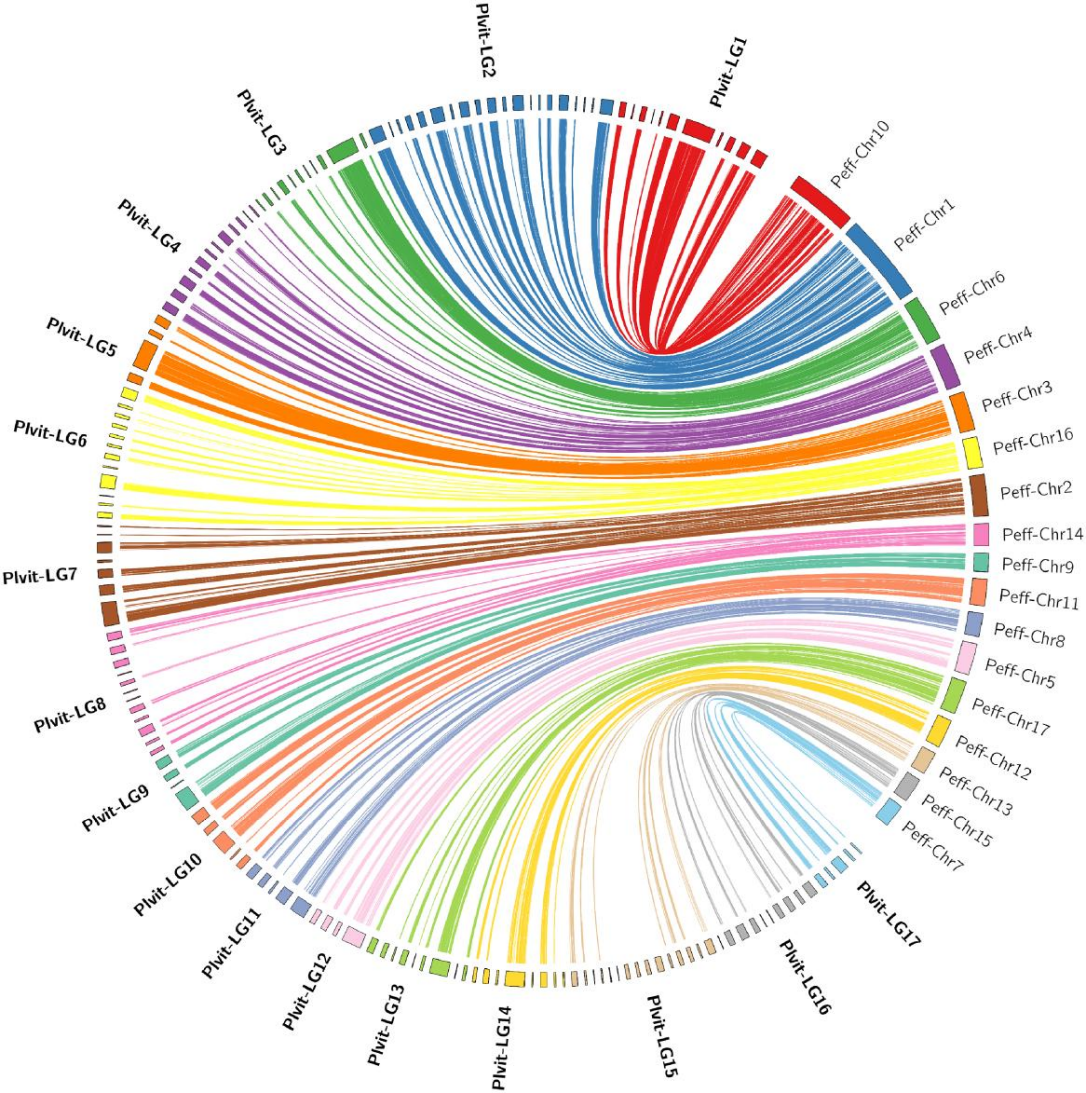
Synteny between the grapevine and spinach downy mildew genomes. On the left, *P. viticola* scaffolds are regrouped and oriented according to the linkage maps and correspond to 87.8% of the genome length (Table 2). On the right are represented the 17 *P. effusa* chromosomes. Each line joins the positions of matching coding sequence (6,365 CDS). Nonanchored scaffolds (not shown) did not display synteny with multiple *P. effusa* chromosomes. The plot was made using Circos v.0.69.9 (Krzywinski *et al.* 2009).

The 154 anchored *P. viticola* scaffolds were aligned with the chromosome-scale assembly of *P. effusa*, the spinach downy mildew pathogen (Fig. 4). Coding sequences from scaffolds in the same LG mapped consistently to the same *P. effusa* chromosome, indicating a high level of synteny between the 2 genomes.

### Pseudoassembly of the genome enables the study of the recombination landscape

Thanks to the linkage data, scaffolds of the reference could be assembled into 17 pseudochromosomes.

#### The recombination landscape is nonuniform

**Fig. 5.**
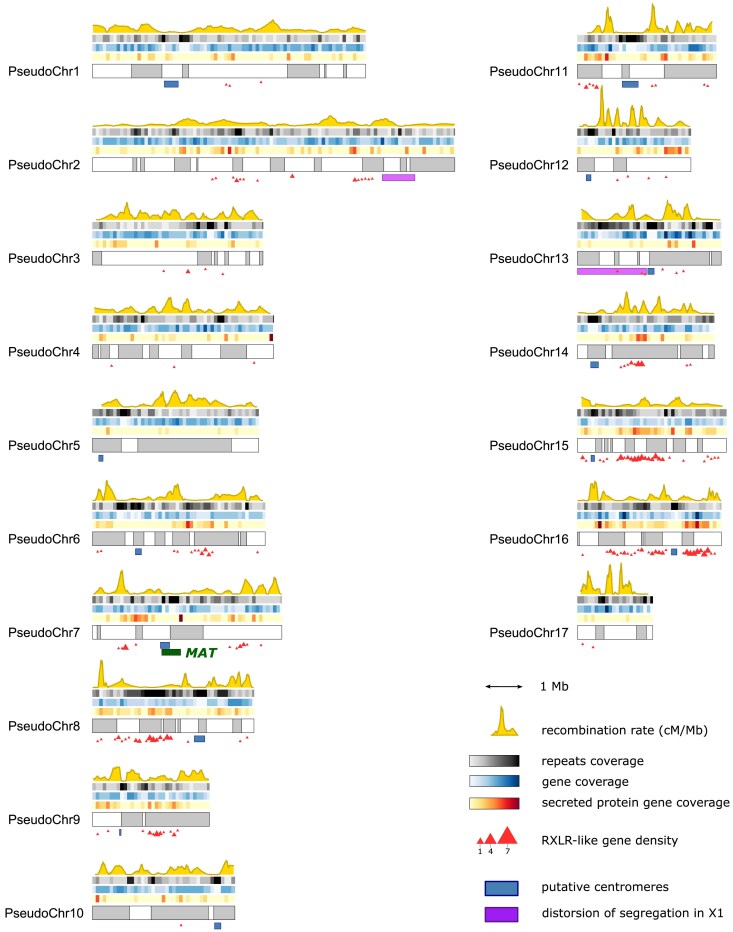
Recombination landscape and genomic features along the reconstructed pseudochromosomes of *P. viticola*. The switch between gray and white sections signals the alternation between scaffolds. On top of the pseudochromosomes is shown, from the top to the bottom: the fraction of bases covered by repeats, genes, and secreted protein genes (nt/kb), all calculated on 100-kb windows. The yellow curve represents the recombination rate in centimorgan per megabase, estimated from the consensus linkage map. Under the pseudochromosomes are displayed notable genomic regions: putative centromeres (in blue), regions affected by segregation distortion in X1 (in purple), and the mating-type locus overlapping with the centromeric region of the pseudochromosome 7 (in green). The density of RXLR-like genes, as defined in Dussert *et al.* (2019), is represented by red triangles. Plot made using r/karyoploteR v1.20.3 (Gel and Serra 2017).

**Fig. 6.**
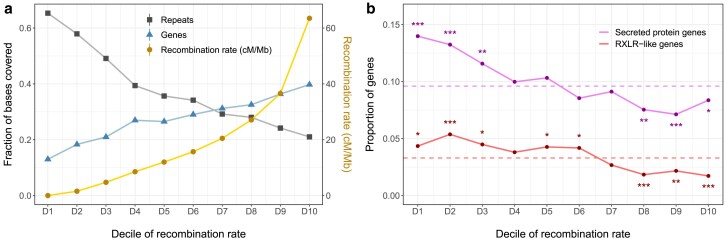
a) Coverage by genes and repeats in each decile of recombination rate. *x*-axis: 10-kb genomic bins grouped by deciles of increasing recombination rate in the consensus *P. viticola* linkage map. Right *y*-axis: average recombination rate (cM/Mb) in each decile. Left *y*-axis: fraction of bases covered by repeated or coding sequences in each decile. b) Enrichment and depletion of secreted protein and RXLR-like genes in each decile of recombination rate. Dashed lines signal the genome-wide proportion of the genes of interest. Asterisks signal significant enrichment or depletion in each decile (hypergeometric test, *P < 0.05, **P < 0.01, ***P < 0.001).

The genetic length of the LGs and the physical length of the corresponding pseudochromosomes are highly correlated (*r* = 0.88 for the consensus map, *P* < 2.2e^−16^). In the consensus map, the average physical distance between markers was 17.7 kb, and the median distance was 8.3 kb. To study the recombination landscape, the recombination rate was estimated in 10-kb bins. It fluctuated importantly along all chromosomes (Fig. 5), from 0.0127 cM/Mb on average in the 10% least recombining bins to 63.5 cM/Mb on average for the top 10% (Fig. 6a). Marker density was calculated every 10 kb in 100-kb sliding windows, and no correlation was found with the recombination rate (*ρ* = −0.03). The recombination rates across windows were well correlated between the parental and consensus maps (*ρ* between 0.62 and 0.72, *P* < 2.2e^−16^), which is consistent with a shared recombination landscape between strains. In all 10-kb bins, the percentage of bases covered by genes and repeats was calculated. The recombination rate was positively correlated with gene coverage (*ρ* = 0.32, *P* < 2.2e^−16^), while the correlation with repeat coverage was negative (*ρ* = −0.39, *P* < 2.2e^−16^). These trends were also visible in the comparison between deciles of recombination rate (Fig. 6a).

Lack of recombination could be observed in several segments on most pseudochromosomes (Fig. 5). Some of them were likely to be centromeric regions. Based on the presence of specific repeated sequences, putative centromeres were confidently identified in 13 pseudochromosomes out of 17. As expected, they clearly corresponded to repeat-rich and gene-poor regions (Fig. 5).

#### Putative effectors are relatively depleted in highly recombinant regions

RXLR-like genes were mostly organized in clusters and particularly abundant in some pseudochromosomes, such as PseudoChr8, 15, and 16 (Fig. 5). By contrast, they were absent or very scarce in half of the pseudochromosomes. The positive correlation between gene content and recombination rate at the 10-kb scale disappeared when considering only secreted protein or RXLR-like genes (*ρ* = 0.03 and −0.04, respectively). In fact, both secreted protein and RXLR-like genes were significantly more abundant than expected in the 3 less recombining deciles of the genome and significantly depleted in the 3 most recombining ones (hypergeometric test, *α* = 0.05) (Fig. 6b).

#### The mating-type locus is pericentromeric

The 570-kb MAT locus overlaps with the centromere of the pseudochromosome 7 (Fig. 5). The entire locus showed little to no recombination, with different behaviors depending on the mating type. In the P2 parent Pv412_11 (MAT-a/MAT-a), 2 COs were detected in the offspring. By contrast, no recombination took place in P1 parents (MAT-a/MAT-b) that possess 2 divergent alleles at the locus.

### Karyotypic anomalies are found in both progenies

#### Aneuploidies and triploidy originate quasi-exclusively from male gametes

**Fig. 7.**
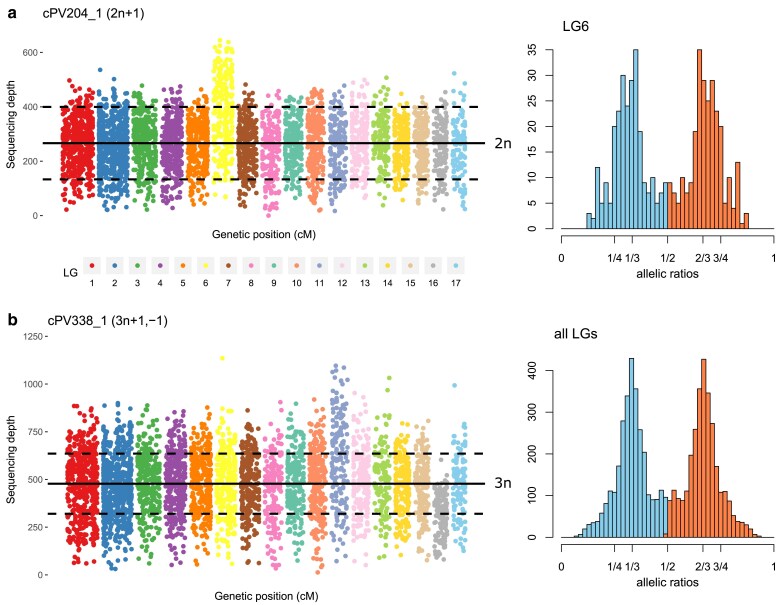
Examples of karyotypic anomalies in *P. viticola* offspring. a) On the left is represented the read depth of all markers across all LGs for cPV204_1, a trisomic individual carrying an extra copy of LG6. The full line represents the average sequencing depth, and the dashed lines are plotted at +50 and −50% of this value. On the right, allelic ratios of heterozygous markers for LG6 show peaks at 1/3 and 2/3 (minor allele in blue, major allele in red). b) Same data for cPV338_1, a triploid individual displaying additional aneuploidies: 4 copies of LG11 (tetrasomy) and 2 copies of LG16 (disomy). On the left panel, dashed lines are plotted at +33 and −33% of the average depth. On the right panel, allelic ratios of heterozygous markers across the entire genome are consistent with a global ploidy number of 3. Additional examples are available in Supplementary Figs. 6–8.

Atypical karyotypes were identified by combining read depth data and allelic ratios for the markers (Fig. 7). These data were consistent with the abnormal phase switches observed in the parental linkage maps. Both progenies exhibited similar levels of aneuploid individuals (1.9% in X1, 2.1% in X2) and triploids (3.2% in X1, 4.3% in X2). Aneuploidies affected various LGs, but their limited number made it difficult to draw conclusions on the frequency at which each LG was concerned (Table 3). Some triploids in X2 displayed additional aneuploidies (Supplementary Fig. 6), as exemplified in Fig. 7b. In diploids, trisomic individuals were the most common, but 1 monosomic strain was identified as well (Supplementary Figs. 7 and 8). Moreover, partial deletion or duplication of chromosome ends was also detected and spanned large segments (>1 Mb) (Supplementary Fig. 8).

**Table 3.** Diploid offspring affected by karyotypic anomalies.

Cross	Strain	Origin of the anomaly	Male parent	Anomaly	Affected linkage group	Approximate length
X1	cPV57_1	Pv412_11	Pv412_11	Trisomy	15	—
X1	cPV101_1	Pv412_11	Pv412_11	Partial deletion	8	1 Mb
X1	cPV138_1	Pv412_11	Pv412_11	Trisomy	16	—
X1	cPV204_1	Pv412_11	Pv412_11	Trisomy	6	—
X1	cPV236_1	Pv412_11	Pv412_11	Trisomy	17	—
X1	cPV243_1	Pv412_11	Pv412_11	Partial duplication	2	1 Mb
X2	cPV308_1	Pv412_11	Pv412_11	Trisomy	17	—
X2	cPV329_1	Pv412_11	Pv412_11	Partial deletion	11	1.4 Mb
X2	cPV409_1	Pv412_11	Pv412_11	Trisomy	3	—
X2	cPV419_1	Pv412_11	Pv412_11	Partial deletion	8	1.5 Mb
X2	cPV462_1	Pv412_11	Pv412_11	Monosomy	16	—

Strikingly, all these anomalies were almost systematically transmitted via the male parent (Tables 3 and 4). There was only one instance of an extra chromosome inherited from the female gamete (LG12 of the triploid cPV479_1). Notably, simple aneuploids in both progenies resulted exclusively from unbalanced male gametes produced by the common parent Pv412_11. All partial deletions and duplications also originated from this strain.

**Table 4.** Triploid offspring and their diverse mechanisms of origin.

Cross	Strain	Origin of the extra chromosome set	Male parent	Additional aneuploidy^a^	Reduced pericentromeric markers (%)	Nonreduced pericentromeric markers (%)	Most likely origin of triploidy
X1	cPV33_1	Pv412_11	Pv412_11		77	23	Dispermy
X1	cPV34_1	Pv1419_1	Pv1419_1		42	58	Dispermy
X1	cPV74_1	Pv412_11	Pv412_11		0	100	Nondisjunction in meiosis I
X1	cPV79_1	Pv412_11	Pv412_11		69	31	Dispermy
X1	cPV89_1	Pv412_11	Pv412_11		38	62	Dispermy
X1	cPV213_1	Pv412_11	Pv412_11		100	0	Nondisjunction in meiosis II
X2	cPV305_1	Pv412_11	Pv412_11		100	0	Nondisjunction in meiosis II
X2	cPV309_1	Pv412_11	Pv412_11	Tetrasomy LG14	100	0	Nondisjunction in meiosis II
X2	cPV313_1	Pv412_11	Pv412_11		46	54	Dispermy
X2	cPV338_1	Pv2543_1	Pv2543_1	Tetrasomy LG11; disomy LG16	100	0	Nondisjunction in meiosis II
X2	cPV447_1	Pv2543_1	Pv2543_1		50	50	Dispermy
X2	cPV449_1	Pv2543_1	Pv2543_1		33	67	Dispermy
X2	cPV479_1	Pv2543_1	Pv2543_1	Tetrasomy LG12 and LG17	100	0	Nondisjunction in meiosis II

^a^All additional aneuploidies originated from male gametes as well, except in the case of LG12 in cPV479_1, for which both parents transmitted 2 chromosome copies.

#### Multiple mechanisms, including dispermy, lead to triploidy

All parent strains were found to occasionally transmit an extra set of chromosomes via male gametes, resulting in triploid offspring (Table 4). To elucidate the mechanism causing this phenomenon, we took advantage of the information available from pericentromeric markers. Thus, we deduced which alleles were transmitted by the male parent for each of the 13 LGs in which a putative centromere was reliably identified (Fig. 5). Briefly, the pattern of reduction or nonreduction to homozygosity of these markers informed us on the most likely origin of the 2 male chromosome sets (Table 4).

In both crosses, we identified several mechanisms that led to a triploid zygote. In half of all cases, nondisjunction of chromosomes during meiosis led to the production of diploid male gametes. These nondisjunction events occurred over the first meiotic division (consistently nonreduced markers, *N* = 1) or during the second one (consistently reduced markers, *N* = 5) (Table 4). Interestingly, we also observed triploids carrying a mix of reduced and nonreduced markers, which can be explained by dispermy, i.e. fertilization by 2 independent haploid male gametes (*N* = 7). Notably, this last mechanism involved all parent strains (Table 4). Therefore, triploidy was caused by abnormal meiosis as well as atypical fertilization.

Finally, some triploids displayed additional aneuploidies in X2. By checking the allelic ratio of variants homozygous in both parents, we found that the female gamete likely transmitted a balanced number of chromosomes in all but one case mentioned in the paragraph above (Table 4).

#### Some individuals present copy-neutral loss of heterozygosity

In total, 4 individuals displayed long runs of marker homozygosity without evidence of partial deletion of the chromosome (Table 5). This loss of heterozygosity (LOH) likely originated from the conversion of the chromosome segment to the other haplotype present in its homologous counterpart, which necessarily occurred postfertilization. Two strains presented LOH in almost the entire LG15. The interval corresponds to the entire long arm of the chromosome, which is acrocentric according to the putative centromere position (Fig. 5).

**Table 5.** Offspring showing LOH on chromosomal segments.

Cross	Strain	Lost haplotype	Affected linkage group	Approximate length
X1	cPV108_1	Pv412_11	12	320 kb
X1	cPV222_1	Pv1419_1	7	730 kb
X2	cPV394_1	Pv412_11	15	3.7 Mb
X2	cPV457_1	Pv412_11	15	3.7 Mb

## Discussion

We established a high-density consensus linkage map of the *P. viticola* genome, allowing us to study recombination activity and its variation across the genome. Thanks to the linkage data, we generated a pseudoassembly of the reference genome, making it possible to conduct analyses at the chromosome scale. This work provides valuable insights on the relation between recombination and architecture of oomycete genomes. To our knowledge, it is also the first unequivocal description of polyploidy in a downy mildew pathogen.

### Chromosome-scale pseudoassembly

Obtaining contiguous assemblies for highly heterozygous oomycetes remains challenging. For such species, linkage maps can still bring decisive inputs. Based on the identified LGs, scaffolds were assembled into 17 chromosomes, which correspond to the ancestral number of chromosome pairs of downy mildews (Fletcher *et al.* 2022). In total, about 88% of the *P. viticola* genome length could be anchored. For comparison, 89% of the *Phytophthora infestans* genome was assembled into chromosomes using similar linkage data in addition to an optical map (Matson *et al.* 2022). In our case, unplaced sequences are small repeat-rich scaffolds on which markers were difficult to define in our setting. Some of them probably correspond to centromeric regions, as we could not reliably position the centromeres in 4 out of 17 chromosomes. Interestingly, we observed a seemingly perfect macrosynteny with the telomere-to-telomere assembly of *P. effusa*, which was also noted between other recent highly contiguous downy mildew genomes (Fletcher and Michelmore 2023). The order of scaffolds determined using the linkage map was congruent with the order inferred by synteny. Thus, high-quality reference genome assemblies could be used to guide assemblies of nonmodel downy mildew species by taking advantage of the high level of synteny between Peronosporaceae (Seixas *et al.* 2021).

### Collinearity and compatibility between parental genomes

The genomes of the 3 parent strains are highly collinear, indicating that structural variations are probably limited in European populations of *P. viticola*. It remains possible that some recombination coldspots were caused by heterozygous inversions that prevent COs in the region and thus may not be detected in a F1 progeny (Stevison *et al.* 2011).

The vast majority of the markers exhibited expected Mendelian segregation ratios along the entire genome. However, 2 regions in different LGs were affected by a significant segregation bias in the first progeny only, despite a common parent between the 2 crosses. Each region was affected independently in each parental map.

In Europe, *P. viticola* populations are weakly structured (Fontaine *et al.* 2013), and our 3 parent strains were collected at similar distances from each other. Thus, the segregation distortions in the cross between Pv412_11 and Pv1419_1 cannot be simply explained by genetic incompatibilities resulting from their distinct geographical origins. The distorted segregation ratios could be caused by a selection bias during the maturation or germination of the oospores. In the case of the distorted loci in Pv412_11, the selection must have been postzygotic because we would have otherwise observed distortion in the 2 crosses. Another possibility would be the presence of meiotic drivers inherited in a non-Mendelian manner. A lot of these selfish elements are sex linked (Taylor and Ingvarsson 2003), but we did not find an association between the distorted loci and mitochondrial markers.

### Crossover frequency and recombination landscape

Among the parent strains, Pv1419_1 exhibited a clearly higher genome-wide recombination rate in both male and female meiosis. Such intraspecific variation is extensively described in many species across the tree of life (Stapley *et al.* 2017). Variation in CO number can also be associated with variation in CO distribution (Ritz *et al.* 2017). In our case, the rise in recombination events in Pv1419_1 is spread across the genome. This could result from a reduced CO interference, which is tightly regulated in plants (Mercier *et al.* 2015) and animals (Zhang *et al.* 2018). A lot of species display different CO rates between male and female gametes, yet to our knowledge no data was available for oomycetes. In our *P. viticola* parent strains, the differences were low, albeit significant, and the trends were contradictory. Lenormand and Dutheil (2005) proposed that different recombination rates between sexes in hermaphroditic organisms could arise from a male–female difference in gametic selection. In oomycetes, meiosis occurs after the encounter of gametangia, leading to a very short haploid phase (Judelson 2009). Gametic selection is thus unlikely to play an important role and promote sex-specific recombination rates.

The similarity of CO distributions between parent strains points to a conserved genome architecture, with close chromosome structures and similar physical constraints during meiosis. Overall, repeat-rich and gene-poor regions were less recombinant, as observed in most eukaryotic genomes (Kent *et al.* 2017). Among oomycetes, a positive association between gene density and recombination was also observed in *P. infestans* (Matson *et al.* 2022).

The clustering of RXLR-like genes on a few chromosomes suggests that they evolved by the expansion of multigenic families. The evolution of effector repertoires through duplication is a common feature of oomycete genomes (Schornack *et al.* 2009). Paralogous effector sequences can indeed rapidly diverge to acquire new functions or escape plant recognition. Meiosis could play an important role in this process because unequal COs can generate new gene copies in effector-rich regions. Thus, frequent COs in effector-rich regions could promote rapid adaptation to host immune responses. In line with this hypothesis, recombination hotspots are enriched in secreted protein and putative effector genes in the genome of several fungal plant pathogens (Croll *et al.* 2015; Laurent *et al.* 2018; Müller *et al.* 2019). However, we found that these types of gene were rather underrepresented in the most recombining windows of the *P. viticola* genome. Indeed, secreted protein genes are preferentially located in repeat-rich regions (Dussert *et al.* 2019) and we showed that these regions tend to recombine less. It has been suggested that the association of effector genes with transposable elements (TEs) in plant pathogens is beneficial for their rapid diversification and a fine-tuning of their expression during infection (Fouché *et al.* 2022). We hypothesize that TE activity may be beneficial to enable a high turnover rate of effectors, but it could partially hinder recombination (Kent *et al.* 2017). A low level of recombination in some clusters of effector genes may also preserve beneficial allelic combinations. In any case, virulence-related genes should not be considered to be consistently associated with highly recombining regions in oomycetes.

### Prevalence and origin of karyotypic anomalies

In our crosses, the majority of the offspring inherited a balanced set of chromosomes from both parents. Aneuploidies (2%) and triploidy (3–4%) are less prevalent than in *P. infestans* crosses, in which as much as 50% of the offspring carry karyotypic anomalies (Matson *et al.* 2022).

An open question remains how much abnormal karyotypes of *P. viticola* contribute to epidemic dynamics in the field. As it turns out, we did not identify any aneuploids in natural strains whose whole genome was sequenced so far, and only one triploid (strain Pv4168_1 from the population studied in Paineau *et al.* 2024). By contrast, *P. infestans* populations worldwide are dominated by a few pandemic clonal lineages, often polyploid (Li *et al.* 2017), and complex levels of aneuploidy are common. Triploidy is also found in the clonal species *Phytophthora cinnamomi* (Engelbrecht *et al.* 2021), and extensive aneuploidy was observed in the progeny of 2 clonal lineages of *Phytophthora ramorum* (Vercauteren *et al.* 2011).

In *P. infestans*, polyploid strains are thought to be fitter for asexual propagation thanks to increased heterosis, but are less fertile during sexual reproduction (Hamed and Gisi 2013). In the case of *P. viticola*, triploid strains may be evolutionary dead-ends if they produce fewer oospores, given that they are the only overwintering form. This probably favors the maintenance of an efficient sexual reproduction that result in a majority of balanced karyotypes. Consequently, the contrasted modes of reproduction between *P. viticola* and the predominantly clonal *P. infestans* could explain their difference in karyotypic stability. It would be interesting to assess the prevalence of triploidy in tropical vine-growing regions where asexual propagation can take place all year round, for example in South East Brazil (Camargo *et al.* 2019; Santos *et al.* 2020).

All but one of the aneuploidies in *P. viticola* arose from uneven disjunction of chromosomes in male meiosis, in sharp contrast with *P. infestans* in which most aneuploidies were found to have an oogonial origin (Hamed and Gisi 2013). One parent (Pv412_11) produced the majority of abnormal male gametes, which suggests that this tendency is subject to intraspecific variation. Strikingly, the extra chromosome sets of triploid offspring were also of paternal origin. Half of them were caused by diploid male gametes. Distinct mechanisms probably exist between female and male meiosis, which lead to a more error-prone meiosis in the latter. Our understanding of the cytological and molecular determinants of gamete formation in oomycetes remains limited, especially so when it takes place inside plant tissues. However, triploidy was also provoked by the fertilization of 1 oogonium by 2 independent haploid male nuclei (dispermy). This provides evidence that triploidy in oomycetes can arise through multiple mechanisms, not exclusively from abnormal meiosis. All 3 parent strains were found to cause dispermy, so it is probably relatively common in nature.

### Self-incompatibility and evolution of the MAT locus

We observed no offspring derived from selfing, confirming that sexual reproduction in *P. viticola* is strictly heterothallic. Secondary homothallism has sometimes been observed in other primarily heterothallic oomycetes, but involved either environmental stress or somatic variation disrupting the mating-type regulation system (Michelmore and Wong 2008; Judelson 2009).

No recombination was observed between the divergent alleles MAT-a and MAT-b of the mating-type locus, in accordance with the strong linkage disequilibrium noted by Dussert *et al.* (2020a). By contrast, recombination can occur in the P2 parent (MAT-a/MAT-a), although it is rare (2 crossover events detected). Since the locus is pericentromeric, the divergence between the 2 alleles may have been favored by the recombination suppression in the vicinity of the centromere. This can explain the maintenance of the asymmetry of heterozygosity that determines the mating type in this species.

The same mechanism of evolution for self-incompatibility loci has been proposed in various eukaryotic kingdoms. For example, complete linkage between the MAT locus and a centromere is observed in some ascomycete or basidiomycete fungi (Idnurm *et al.* 2015). Similarly, sex-determining regions are pericentromeric in several dioecious plants (Charlesworth 2019).

### Adaptation by LOH

Copy-neutral LOH was observed in 4 individuals and affected large segments. This phenomenon has been extensively described in *Phytophthora* spp. (Chamnanpunt *et al.* 2001; Lamour *et al.* 2012; Dale *et al.* 2019). Moreover, isolates of the downy mildew pathogen *P. effusa* can exhibit intermediate LOH affecting only a fraction of the nuclei (Fletcher and Michelmore 2023). Spontaneous LOH due to mitotic recombination could also occur frequently in *P. viticola*, but may remain undetected as long as it stays limited to a minor proportion of the nuclei. In our case, LOH events must have taken place during the first step of strain propagation, as it would otherwise not have affected the entirety of the material we sequenced, and therefore not have been revealed. LOH can provide an additional way of adaptation during the growing season, through the fixation of beneficial alleles faster than annual sexual reproduction. For example, important traits such as fungicide tolerance and virulence are determined by codominant or recessive alleles (Lamour *et al.* 2012; Matson *et al.* 2022; Paineau *et al.* 2024). It has also been shown that LOH can lead to a gain of virulence in *P. effusa* (Lyon *et al.* 2016).

Interestingly, 2 strains in the present study displayed LOH on an identical segment that corresponds to the entire long arm of the acrocentric chromosome 15. This suggests that some chromosomes may be affected by mitotic recombination more often. Similarly, some regions exhibited LOH more frequently in different clonal lineages of *P. ramorum* (Dale *et al.* 2019).

### Conclusion

*Plasmopara viticola* appears to be a conventional example of a highly sexual and self-incompatible organism. It is therefore an interesting plant pathogen model to study the response to new selection pressures such as the deployment of resistant varieties. As the formation of primary inoculum fully depends on sexual reproduction in temperate vine-growing regions, the recombination landscape is likely to have an impact on the efficacy of selection at different loci. The possibility of karyotypic variations should be kept in mind in future research on *P. viticola* genetics. In particular, the potential link between ploidy level and variations in fitness remains a question to be addressed. The linkage maps described here will be useful to guide future de novo chromosome-scale assemblies of *P. viticola* genomes. Moreover, they pave the way for QTL mapping of crucial traits such as virulence toward disease-resistant grapevines.

## Supplementary Material

jkae259_Supplementary_Data

## Data Availability

Allegro probe sequences, VCF files, and linkage map data are available at https://doi.org/10.57745/KX5YAQ. Whole genome Illumina DNA sequences of the parent strains are available at NCBI SRA under project number PRJNA1095879. Allegro targeted sequencing data of the parent strains and their progeny are available at NCBI SRA under project number PRJNA1107130. Supplemental material available at G3 online.
