# Supplementary material for: Recombination landscape and karyotypic variations revealed by linkage mapping in the grapevine downy mildew pathogen Plasmopara viticola
Source: G3 (Bethesda). 2024 Dec 2;15(1):jkae259. doi: 10.1093/g3journal/jkae259 (PMC11979753; doi:10.1093/g3journal/jkae259)
Supplement: jkae259_Supplementary_Data [file jkae259_supplementary_data.pdf]

Supplementary Figures for

Recombination landscape and karyotypic variations revealed by linkage mapping in the grapevine downy mildew pathogen *Plasmopara viticola*

Dvorak E., Mazet I., Couture C., Delmotte F., Foulongne-Oriol M.

This PDF file includes:

Figs. S1 to S8

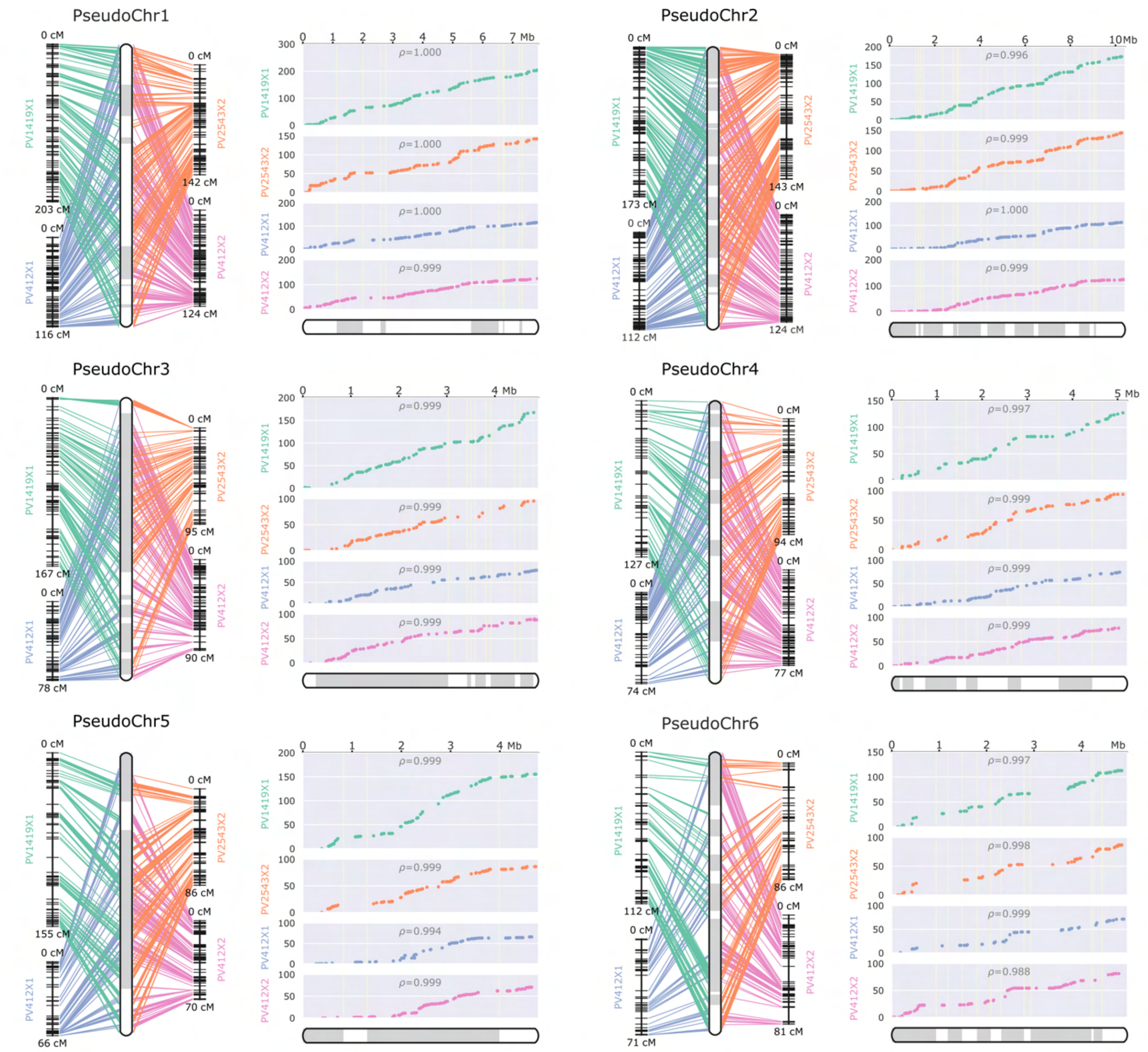

Fig. S1. ***Plasmopara viticola*** **parental linkage maps** (part 1/3)

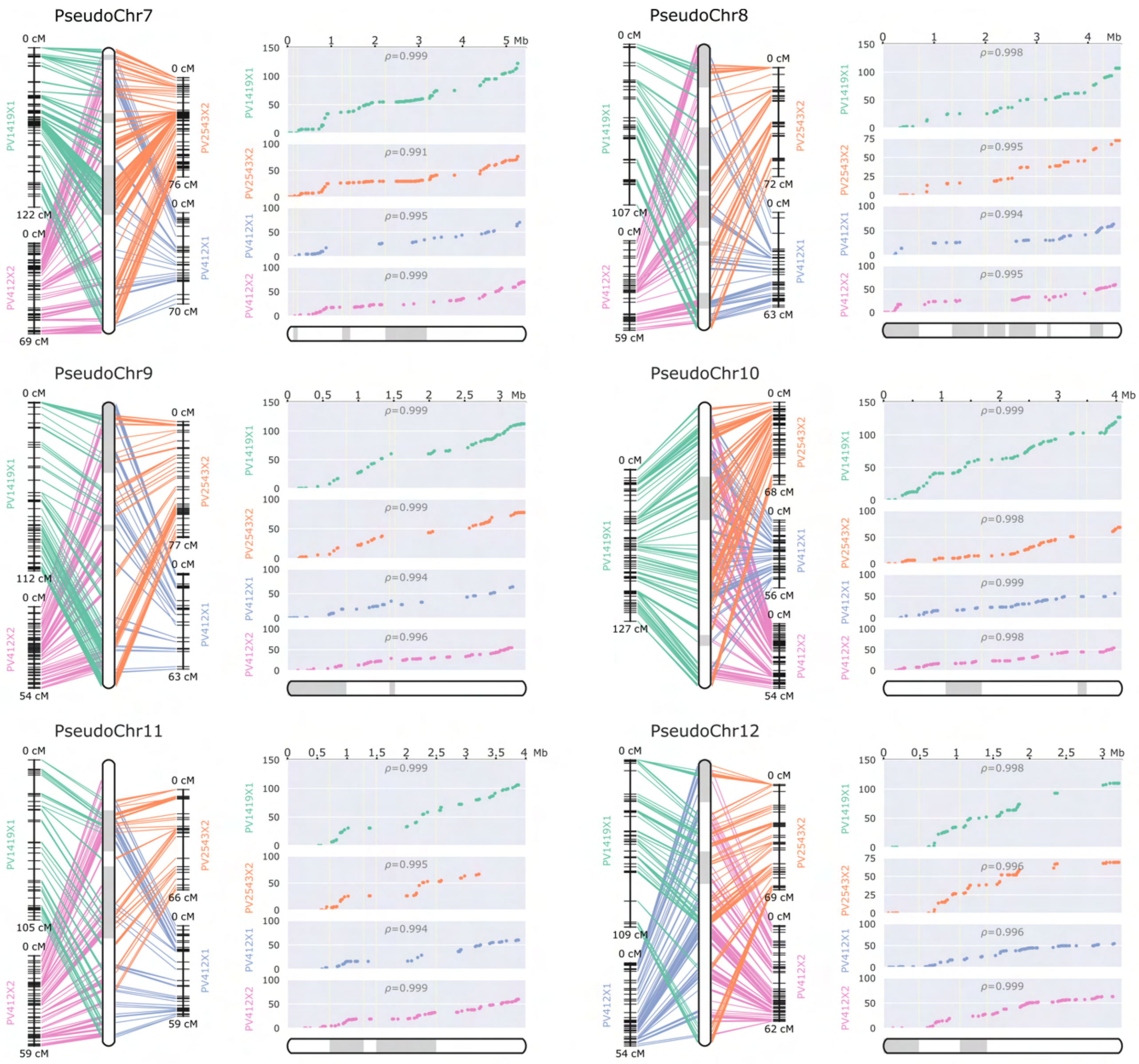

Fig. S2. ***Plasmopara viticola*** **parental linkage maps** (part 2/3)

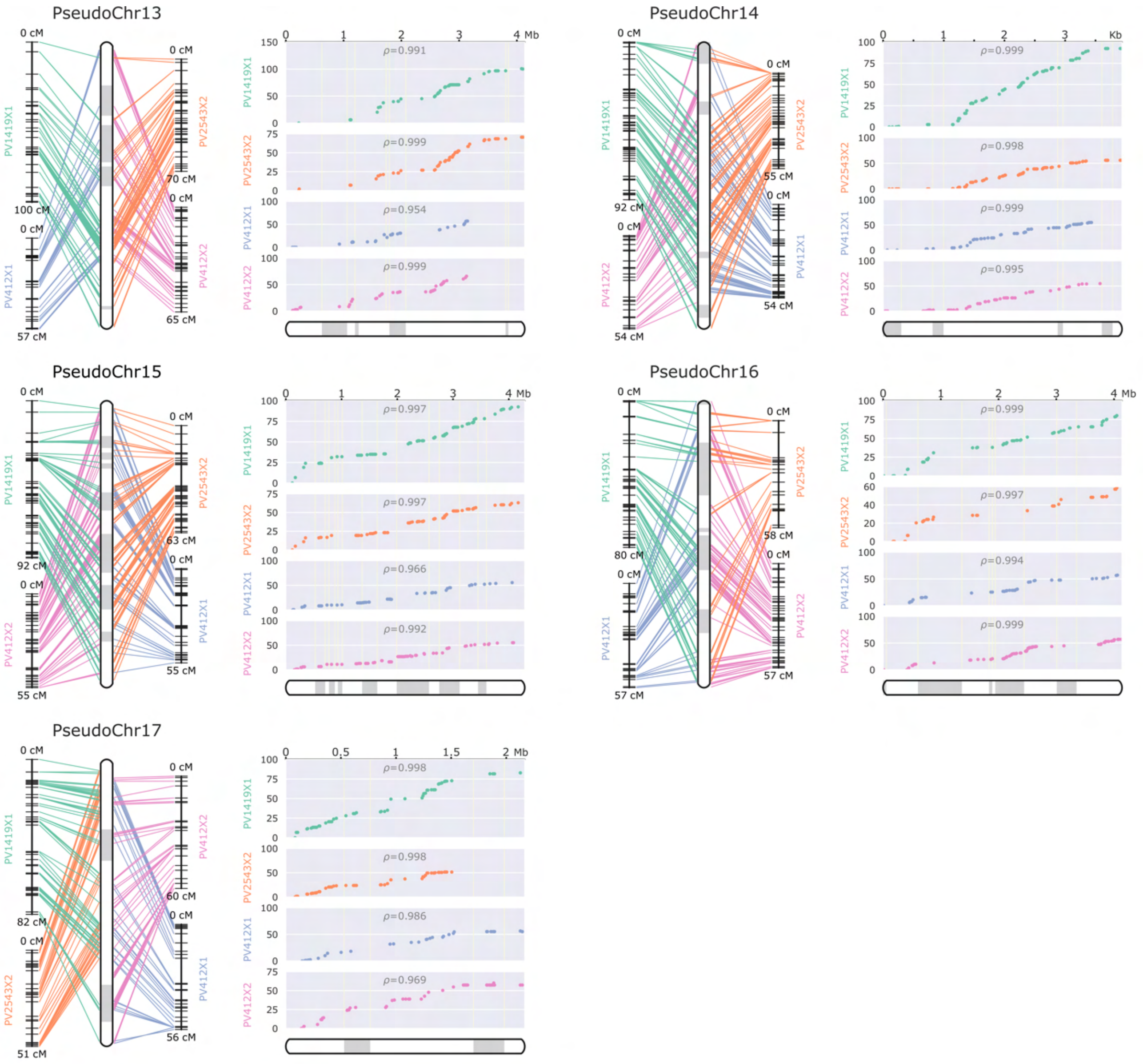

Fig. S3. ***Plasmopara viticola*** **parental linkage maps** (part 3/3)

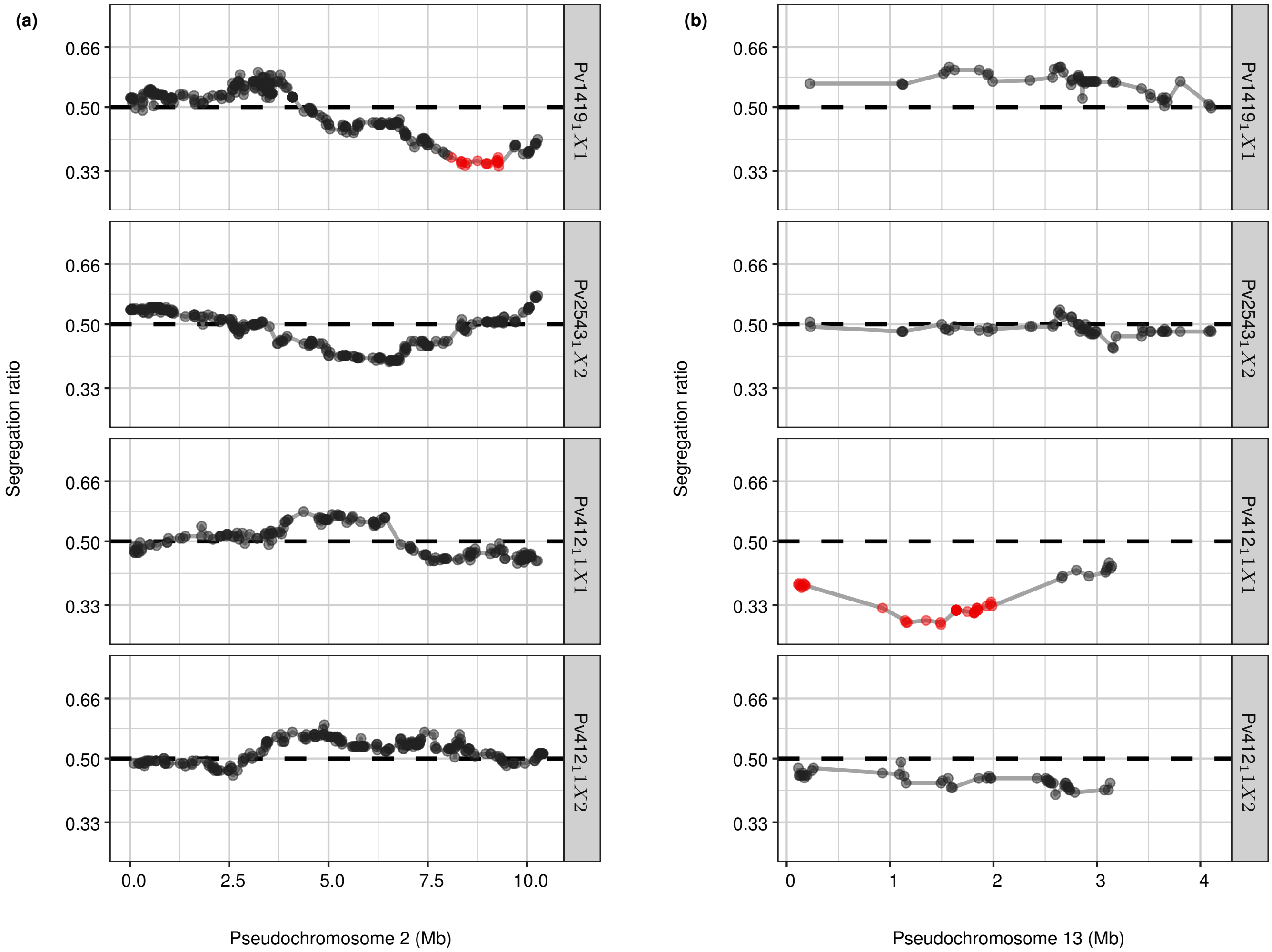

Fig. S4. (a) Segregation ratios of markers along pseudochromosome 2. Red points signal marker significantly distorted according to an adjusted Chi-squared test($\alpha <$ 0.01 / number of markers in the linkage map). Note that the threshold of significativity varies between linkage maps because the total number of markers differs.
(b) Same data on PseudoChr13.

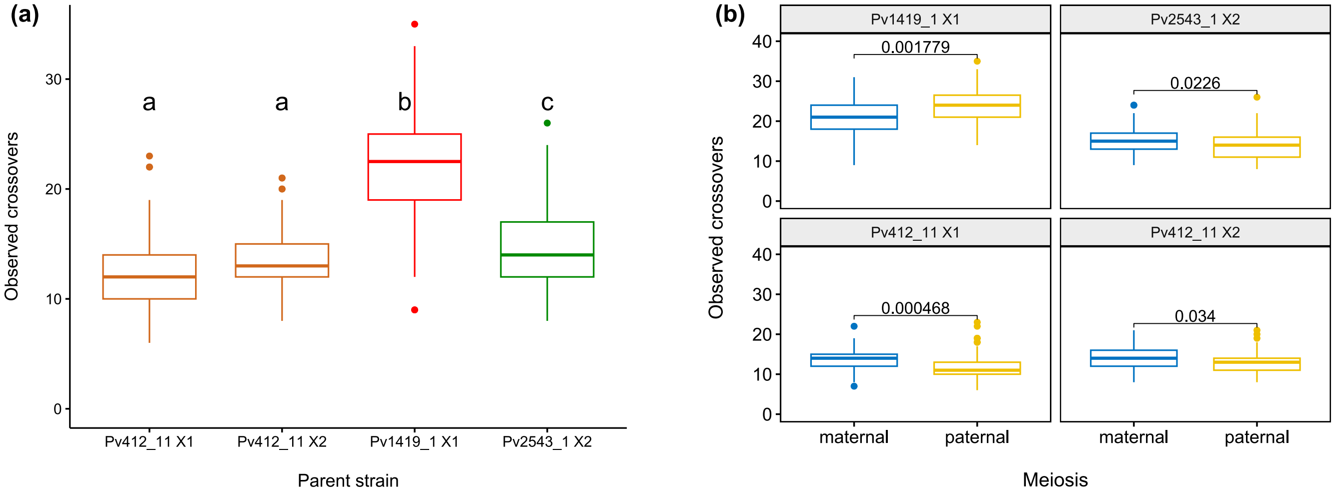

Fig. S5. (a) Number of observed crossovers by individual in each parental linkage maps. Different letters indicate significantly different means according to Tukey's HSD test ($\alpha < 0.05$).
(b) Same data depending on maternal or paternal origin of the gamete. Between box-plots are indicated p-values from two-sample T-tests.

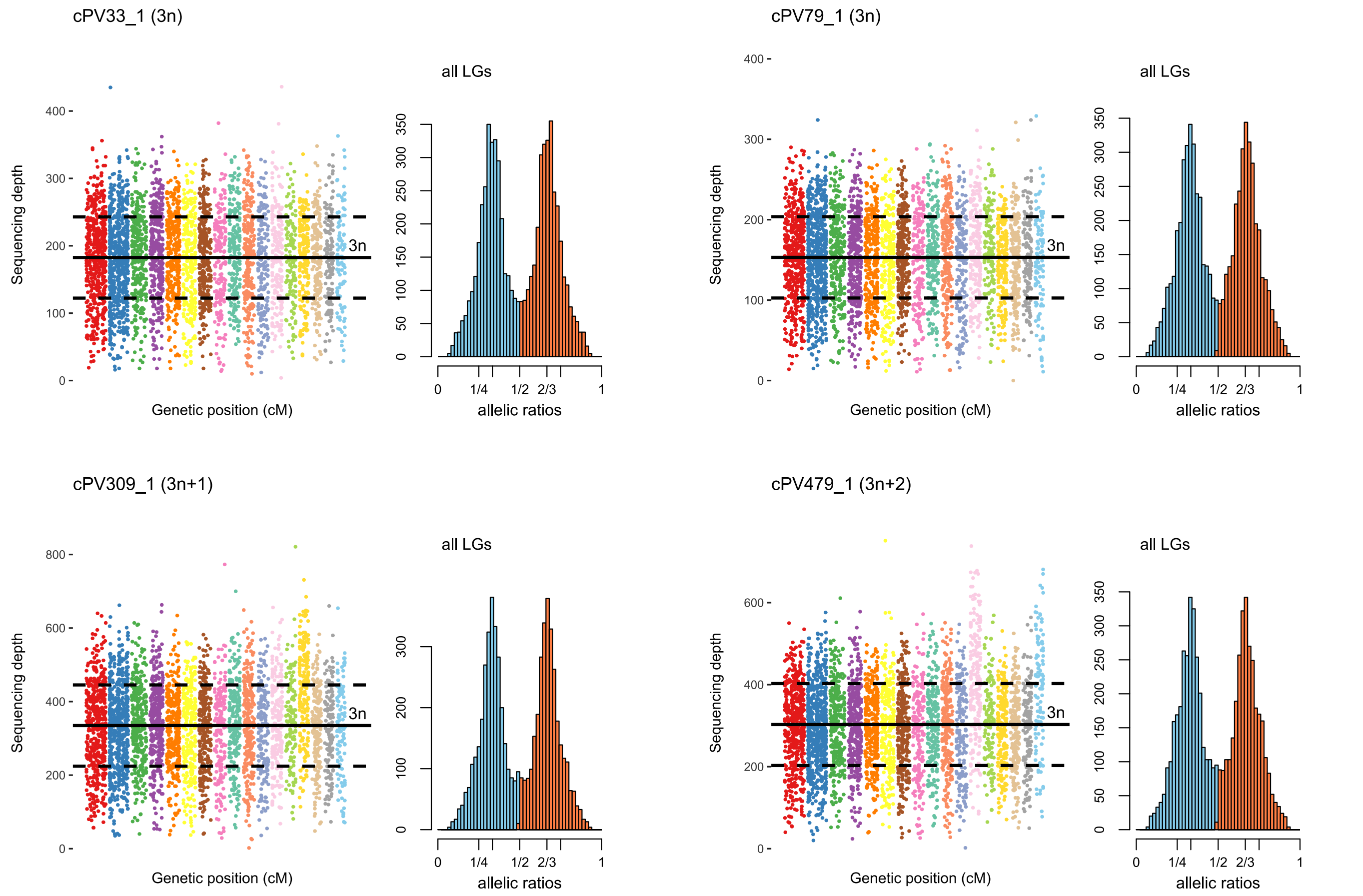

Fig. S6. Karyotypic anomalies in *P. viticola* offspring (part 1/3). Triploid strains. The full line represents the average sequencing depth, and the dashed lines are plotted at +33% and -33% of this value.

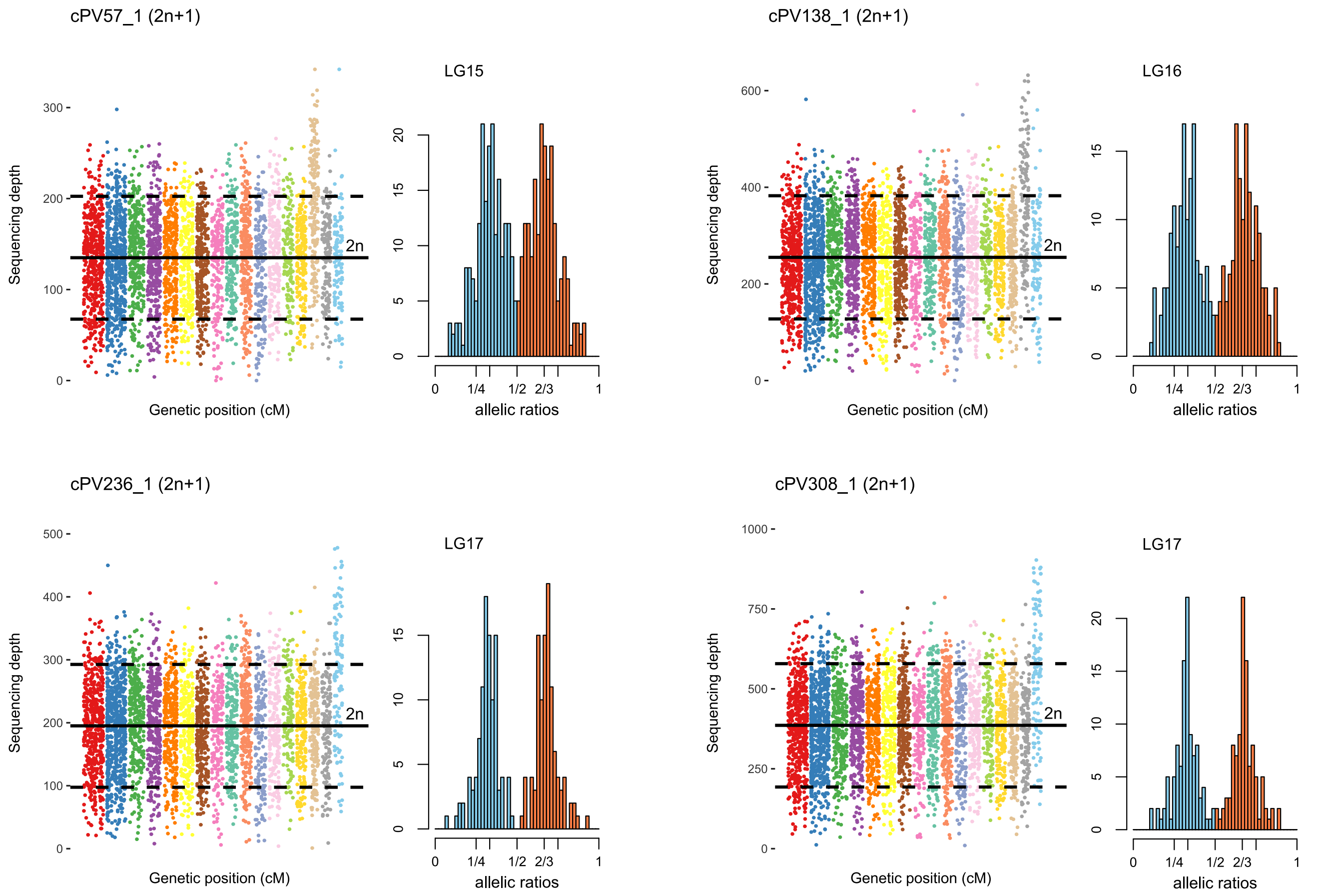

Fig. S7. Karyotypic anomalies in *P. viticola* offspring (part 2/3). Trisomic strains. The full line represents the average sequencing depth, and the dashed lines are plotted at +50% and -50% of this value.

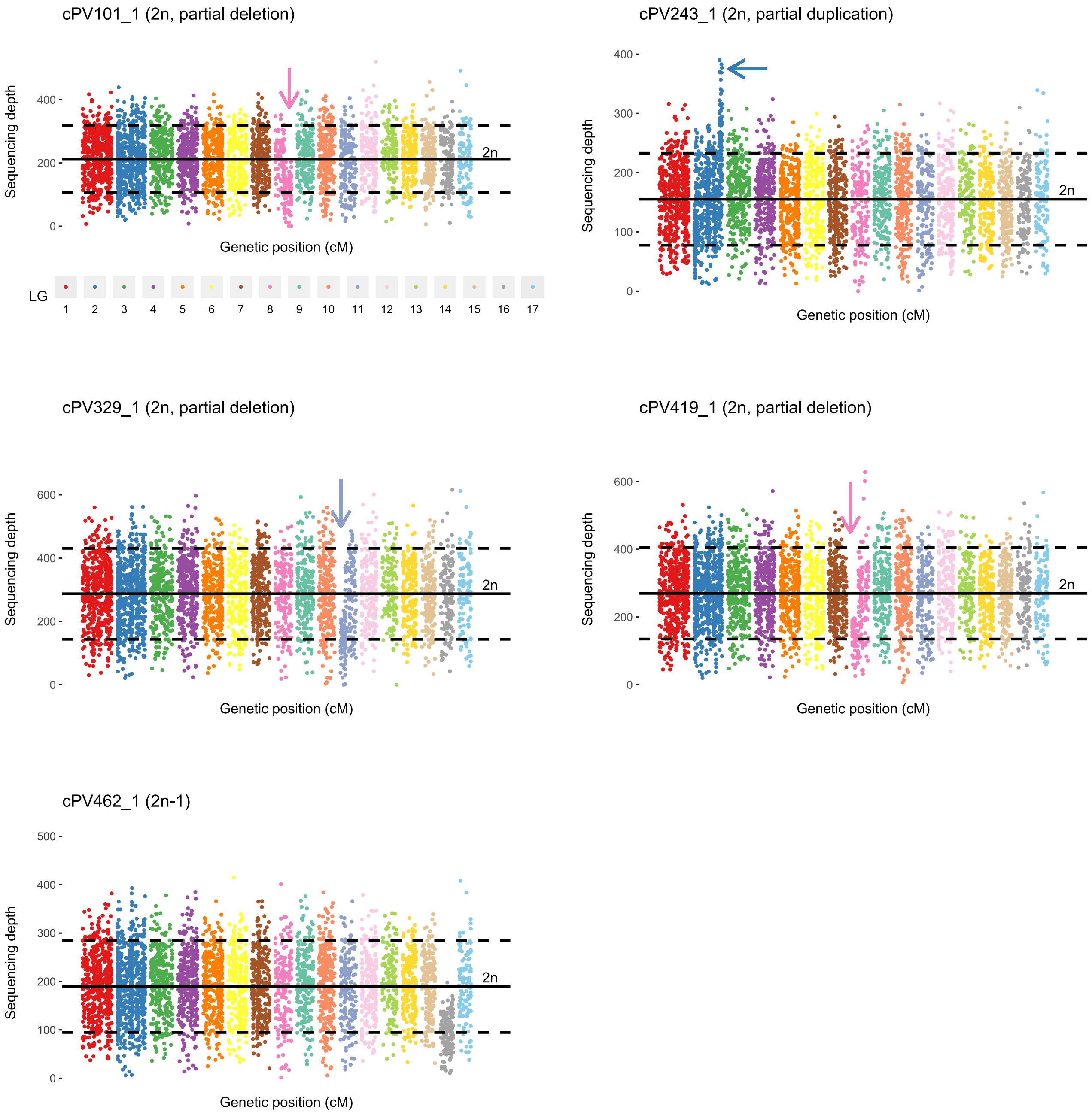

Fig. S8. Karyotypic anomalies in *P. viticola* offspring (part 3/3). Strains affected by partial deletion/duplication and monosomy. The full line represents the average sequencing depth, and the dashed lines are plotted at +50% and -50% of this value. Arrows indicate regions deleted or duplicated in one chromosome copy.
